# CoMAD: a transferable method for building cognition-aware mathematics assessment datasets for educational AI research

**DOI:** 10.1016/j.mex.2026.104071

**Published:** 2026-07-25

**Authors:** Tibakanya Joseph, Joyce Nakatumba-Nabende, Agaba Ezra Joab, Ampeire Henry Kariisa

**Affiliations:** a Department of Computer Science, Makerere University, P. O. Box 7026, Kampala, Uganda; b Department of Networks, Makerere University, P. O. Box 7026, Kampala, Uganda; c College of Education and External Studies, Makerere University, P. O. Box 7026, Kampala, Uganda

**Keywords:** Cognition-aware datasets, Educational AI, Mathematics assessment, Curriculum alignment, Bloom’s taxonomy, Webb’s depth of knowledge, Cognitive annotation, Linguistic features, FAIR data principles

## Abstract

Recent advances in Artificial Intelligence (AI), particularly large language models (LLMs), have accelerated research into automatic item generation, thereby fueling demand for curriculum-aligned, cognitively aware, contextualised datasets. However, there are limited cognitively annotated, contextually grounded, authentic assessment datasets that exhibit linguistic complexity and curriculum-aligned constructs. Moreover, dataset-construction methodologies struggle to integrate established educational taxonomies, linguistic complexity indicators, and FAIR-oriented data management into a single, transparent, and reproducible framework. We present the Cognition-Aware Mathematics Assessment Dataset construction (CoMAD), a transferable, FAIR-oriented framework for creating and curating higher-cognition, curriculum-aligned datasets augmented with computational data engineering. The method is validated using HiCogMath, a mathematics dataset in which cognitive annotations are based on Bloom’s taxonomy and Webb’s Depth of Knowledge, operationalised through Hess’s Cognitive Matrix (CRM). CoMAD is a modular dataset-creation method that is scalable to other subjects, curricula, and contexts, creating datasets suitable for cognitive modelling, data mining, assessment analytics, automated item generation, Knowledge graph construction, and LLM alignment.

• Transferable framework for construction of cognition-aware assessment datasets with curriculum alignment and linguistic profiling.
• Presents transparent quality assurance for enhancing reliability, replicability and reusability of datasets.
• Provides a modular framework that is transferable to diverse curricula, domains and contexts via annotation protocols.

## Graphical abstract

**Figure:**
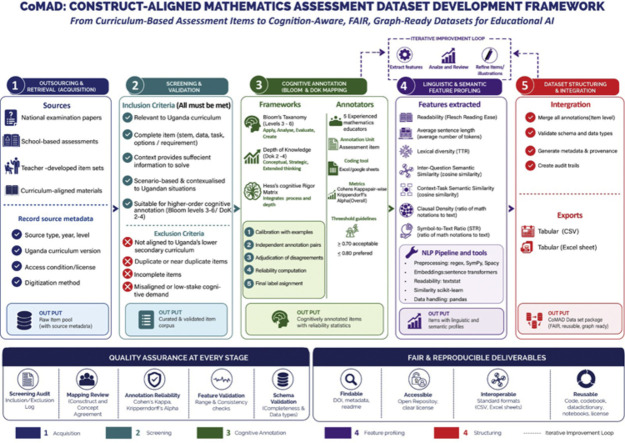

Specifications table

**Table:** 

**Subject area**	Computer Science
**More specific subject area**	Cognition Modelling, Data Mining, Assessment Analytics, Automated Item Generation, Knowledge Graph Construction, and LLM Alignment.
**Name of your method**	Cognition-Aware Mathematics Assessment Dataset construction (CoMAD)
**Resource availability**	Repository: Mendeley Data Data identification number: 10.17632/vb334s8cmn.2 Direct URL to the data: https://data.mendeley.com/datasets/vb334s8cmn/1 Repository Name: HiCogMath: A Cognition-Aligned Dataset for Higher-Order Competency-based Mathematics Assessment

## Background

Mathematical datasets have become critical resources for evaluating large language models, yet most benchmarks measure mathematical reasoning rather than support the modelling of educational assessments. For instance, Cobbe et al. developed the GSM8K dataset for analysing grade-school mathematical reasoning through word problems [1]. Another solution-generating, higher-order reasoning dataset was created by Hendrycks et al., [2]. LILA also combined 23 mathematical reasoning tasks of varying complexity to create another dataset [3]. The most recent ones, such as MathVista [4], CMM-Math [5], and MMMU [6], which are multimodal, extend mathematical reasoning through visual contexts.

While these datasets are valuable for LLM evaluation, they do not directly model mathematics assessment constructs, specific curriculum alignment or cognitive demands in the context of educational measurements where assessment items are designed to elicit evidence of learning. Thus, it is critical to distinguish benchmark difficulty from cognitive complexity. Whereas benchmark difficulty is based on model-specific indicators such as accuracy, these do not demarcate the cognition that an assessment item intends to activate. This is because educational assessment aims to measure learners' competencies by aligning them with intended learning outcomes and defensible constructs [7]. While the current reasoning dataset does not operationalise constructive alignment, educational assessment standards require connection among intended learning outcomes, learning experiences and assessment tasks. As a result, they are short in modelling curriculum intent, construct validity, and cognitive demand.

A more fundamental gap lies in the limited methodological approaches for developing cognition-aware datasets, which would expand educational AI research beyond performance evaluation toward learner modelling, adaptive assessment, automated item generation, and educational retrieval systems. Existing approaches rarely attempt to operationalise cognitive complexity, particularly through established cognitive taxonomies. For instance, Bloom’s revised taxonomy characterises cognitive processes, whereas Webb’s Depth of Knowledge (DoK) [8] describes the depth of reasoning required by a task [9]. Some frameworks like Hess’s Cognitive Rigor Matrix (CRM) [10] integrates these cognitive perspectives in a systematic integrative manner; incorporating such firm frameworks into the construction of AI-ready datasets remains limited. Notably, even the established cognitive taxonomies are not self-executing labels; they require expert interpretation, calibration, adjudication, and reliability evaluation. When tasks require integrating multiple concepts, cognitive annotations may become inconsistent, making it hard to replicate cognitive modelling.

Mathematics curriculum-aligned datasets are especially scarce in low-resource educational contexts [11]. Moreover, existing methodologies do not provide sufficient guidance for constructing reproducible, cognitively aware, and curriculum-aligned datasets to support educational AI applications across diverse educational systems. To close the gap, we present the Construct-Aligned Mathematics Assessment Dataset Development (CoMAD), a transferable methodological framework whose stages can be re-instantiated in other curricular systems. It is a multistage methodology that integrates curriculum mapping, cognitive annotation, reliability adjudication, linguistic profiling, metadata standardisation, and FAIR-oriented data packaging. We validate CoMAD by developing HiCogMath, a higher-order, curriculum-aligned mathematics assessment dataset derived from Uganda’s lower secondary education system. Thus, the contribution of this work is not HiCogMath itself, but rather the replicable method for constructing cognition-aware educational datasets that support educational assessment modelling, cognitive complexity analysis, educational LLM alignment, knowledge graph development, and future AI-driven assessment research across diverse educational contexts.

## Method details

**Fig. 1:**
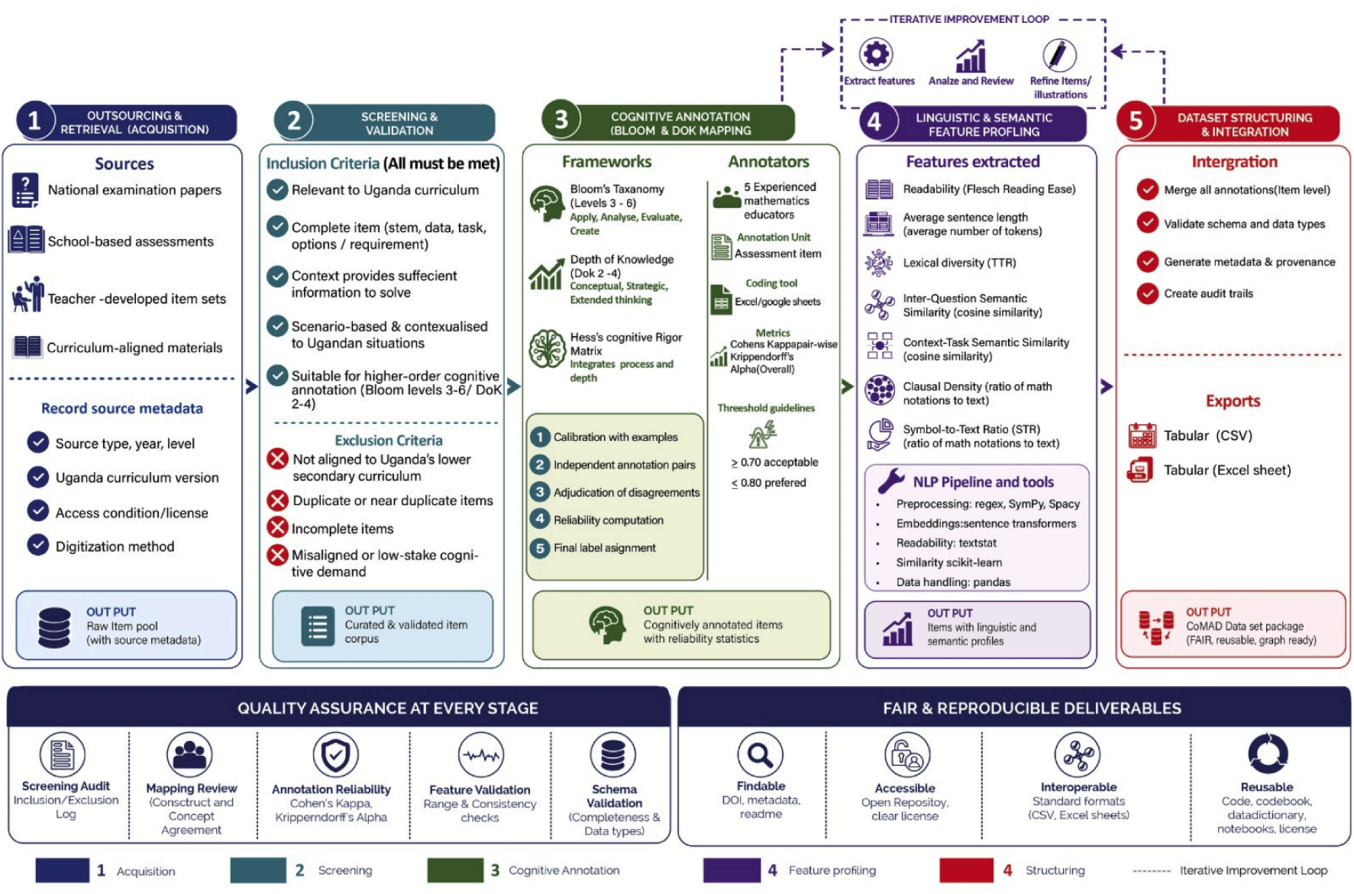
CoMAD methodological framework.

**Fig. 2:**
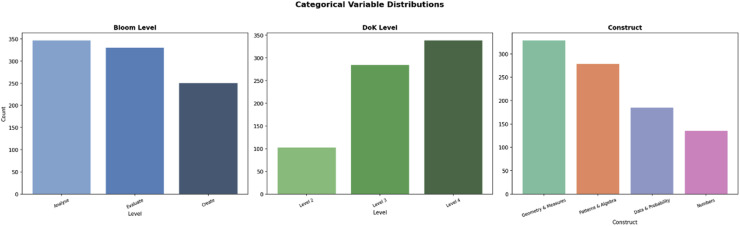
Distribution of categorical variables of the dataset.

CoMAD is a multistage framework designed to systematically create reusable AI datasets for educational assessment research. It supports the transformation of domain-specific assessment materials such as mathematics into structured datasets while ensuring constructive alignment, cognitive complexity, conceptual structure, and linguistic features suitable for diverse use cases. CoMAD follows a systematic curation and annotation approach comprising five steps: sourcing, screening and eligibility validation, expert curation and annotation, linguistic profiling, and structuring, as shown in Fig. 1. To improve consistency, replicability and generalisation across educational levels, curricula and context, each stage involves quality assurance procedures.. As illustrated in Fig. 2, the human annotation process resulted in a dataset of 929 items, categorised according to their respective Bloom, DoK, and construct-level distributions.

The initial stage of dataset creation involved collecting items from readily accessible open-access materials in secondary school examination archives and from publicly available school-based assessment materials crafted by expert teachers and validated against predefined screening criteria. Next, cognitive and curriculum alignment annotation was performed by expert mathematics annotators who are item writers and have taught in Ugandan schools for >10 years. They edit items, map curriculum alignment, conduct exclusion and inclusion and annotate cognitive labels to the retained items that are scenario-based, assessing higher cognition at Bloom’s Taxonomy levels [9] Analyse, Evaluate, and Create, and Webb’s Depth of Knowledge [8] Skills and Concepts, Strategic Thinking, and Extended Thinking levels 2 to 4, consistent with Hess’s Cognitive Rigour Framework [10]. The annotation took place using an independent paired-review design, with the last person serving as a tiebreaker. Moreover, throughout the process, tiebreakers were not treated as unquestionable authorities and were rotated in each session; disputed cases were reviewed against our explicit annotation protocol. Reliability was quantified using Cohen’s Kappa [12] for pairwise annotations and Krippendorff's alpha [13] for overall agreement as presented in Table 4.

**Fig. 3:**
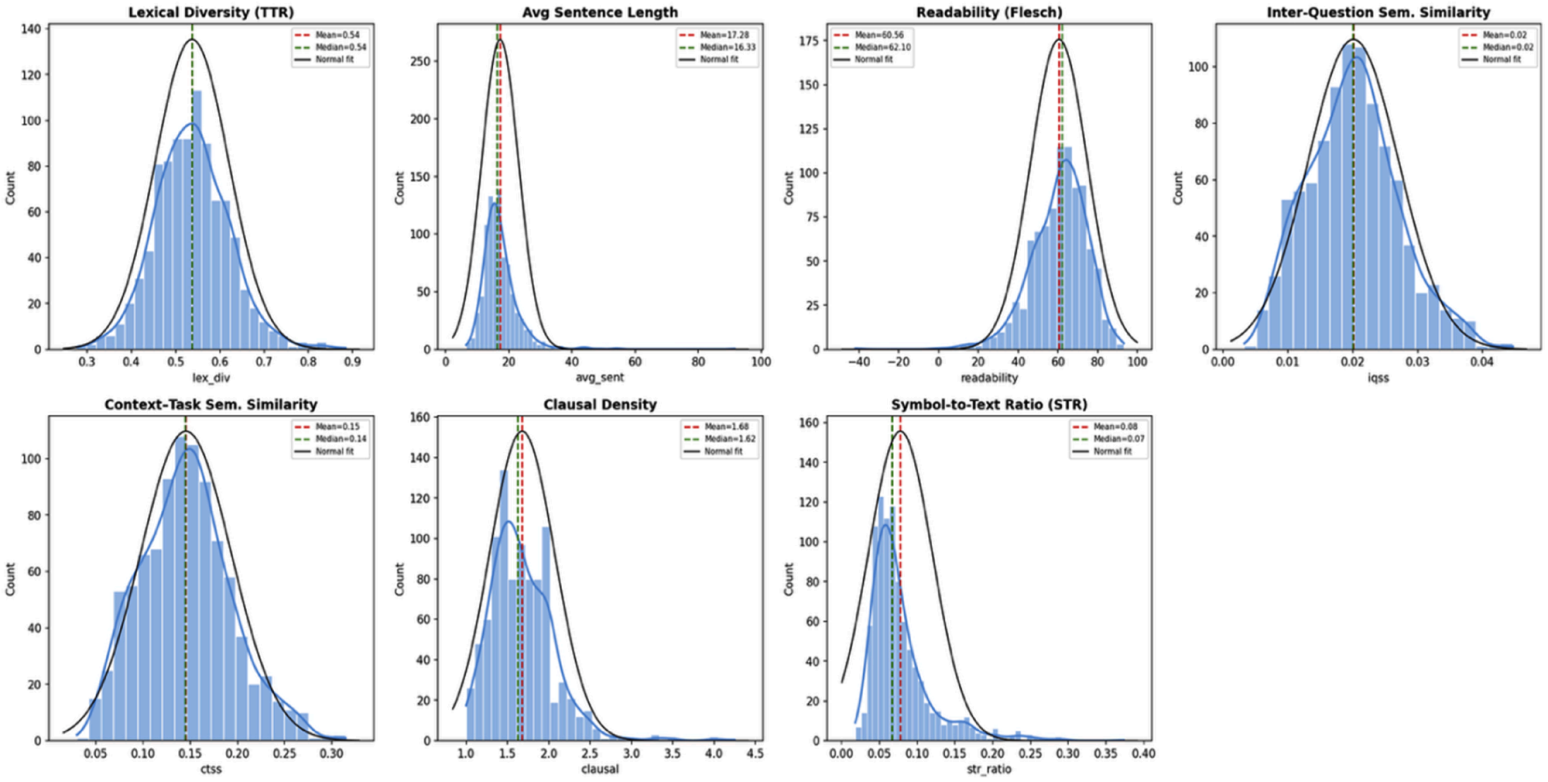
Average values for all derived features.

Later, each included item underwent automated extraction of linguistic and structural features via a natural language processing pipeline. This stage involved text normalisation, tokenisation, segmentation, and parsing of mathematical symbols to enable the processing of mathematical expressions. We then computed various linguistic features, including Lexical Diversity, derived using the Type-Token Ratio [14]; Average Sentence Length; Readability, derived from Flesch Reading Ease Scores [15]; and Clausal Density. Moreover, we employed embedding-based methods [16] to derive semantic analyses, such as Inter-Question Semantic Similarity and Context–Task Semantic Similarity, to analyse structural and semantic connections among items. Fig. 3 shows combined histograms, with Mean values also presented in Table 5 for the derived features. Finally, we computed the density of mathematical expressions relative to the amount of text per item using the Symbol-to-Text Ratio (STR). In the end, 929 items resulted from this rigorous process as presented in Fig. 2 distributions across Bloom, DoK and Construct levels. All annotations, features, and metadata were integrated into a standardised dataset schema that facilitates reuse in replication, cognitive modelling, educational data mining, assessment analytics, large language model training, retrieval-augmented generation systems, and agentic educational AI applications.

### Stage 1: assessment item acquisition and screening

Stage 1 establishes how the corpus of traceable items in Uganda's mathematics assessment specifications was acquired. The purpose of this stage was to obtain items aligned with Uganda’s lower secondary (up to senior four) end-of-cycle competency-based curriculum, thereby establishing a large corpus for subsequent annotation and profiling. The items were acquired from four main sources: (i) publicly accessible national examination papers from Uganda National examination administered for the past two years, and sample papers sent out to guide teachers on assessment, (ii) school-crafted mathematics assessment materials developed by teachers as activities of integration and end-of-year/end-of-term exams, (iii) teacher-developed item sets in public pamphlets, and (iv) curriculum-aligned mathematics assessment items in curriculum documents. These provided authentic assessment practices that reflected real-world expectations and cognitive complexity. For each material source, we recorded the source type, year, education level, curriculum version, ownership, access conditions, and permission status.

All materials were digitised and standardised in tabular structures using Microsoft Excel. We deduplicated by eliminating duplicate items using exact string matching in MS Excel. Using manual methods, we identified and edited semantically similar items since we needed as many items as possible. Later, manual verification was conducted to assess completeness, the transcription of mathematical notation using LaTeX, and the alignment of contextual information.

Using predefined inclusion and exclusion criteria summarised in Table 1, initial screening was conducted. Items were included if they were complete, scenario-based, contextualised to Uganda, assessed concepts taught in Uganda's lower secondary mathematics, and represented higher-order tasks. In this work, we minimised reliance on expert subjective judgement regarding contextual relevance by providing a clear operational definition. For instance, contextual relevance meant that an item reflects Ugandan-recognisable environments, organisations, economic activities, cultural practices, geographic entities, or educational experiences referenced in national curriculum materials. Additionally, a scenario-based item was defined as one embedded in a meaningful, real-world or curriculum-relevant problem situation from which an item taker must identify and apply mathematical knowledge and skills.

**Table 1:** Exclusion and inclusion screening criteria.

Criterion	Include	Exclude
Curriculum Alignment	The item assesses concepts taught in Uganda's mathematics lower secondary curriculum	The item assesses concepts not taught in Uganda's mathematics lower secondary curriculum
Item Completeness	Full context and task are present	Missing components, e.g., concepts, scenarios, or tasks
Scenario-based with tasks	Items with contextualised problems in text form with corresponding tasks.	Items presented in tasks with scenarios, contextualising problems in word form.
Scenario Context	Scenarios presented in Uganda's context	Items whose scenarios are biased to contexts other than Uganda.
Higher-order Cognitive suitability	Potential for Analyse, Evaluate, Create of Bloom’s taxonomy or Webb’s DoK 2–4	Pure recall or other lower levels of the 3 top levels of Bloom’s taxonomy or Webb’s DoK 2–4
Duplication	Unique item to others	Exact string match

Notably, moderate items were improved through minor edits to scenarios, concepts, figures, mathematical expressions and tasks. To allow context, curriculum and cognitive refinement, original and modified item versions were tracked separately. Modified items were assigned to a modification flag specifying how an item was affected, for instance, context, wording, mathematical values, task demand, or construct alignment. Others were discarded if they were extremely low-stakes, duplicates, incomplete, assessed concepts outside the curriculum, purely procedural, or lacked sufficient evidence to determine the complexity level.

All retained items were assigned a unique identifier in the format HiBloom_00 to support traceability throughout subsequent stages.

### Stage 2: curriculum alignment and construct mapping

To ensure curriculum alignment, we conducted stage 2 to link each included assessment item to the curriculum assessment specifications provided by the Uganda National Examination Board, as presented in Table 2. In this work, a construct refers to a broad assessable domain of mathematical competences, while a concept is a more specific mathematical idea, procedure, principle or topic assessed within that construct. Four curriculum constructs were identified: Patterns and Algebra, Data and Probability, Numbers, and Geometry and Measures. In a curricular context, researchers may need to derive equivalent constructs from official assessments, curricula specifications, or competency domains before contextualising a similar mapping protocol. A comprehensive summary of all annotation stages, procedures, and deliverables is provided in Table 3.

**Table 2:** Table of constructs.

No	Construct	Topics
1	Patterns and Algebra	Patterns and Sequences, Equations of Lines and Curves, Algebra, Mapping and Relations, Inequalities and Regions, Linear Programming, Simultaneous Equations, Rectangular Cartesian planes, Loci
2	Data and Probability	Data collection and presentation, Graphs, Set Theory, Matrices, Probability
3	Numbers	Number Bases, Integers, Fractions, Percentages, and Decimals, Numerical Concepts, Ratios and Proportions
4	Geometry and Measures	Geometrical Construction, Bearings, Angle Properties of Geometric Figures, Reflection, Business Mathematics, Time and tables, Similarities and Enlargement, Circles and circle properties, Rotation, Length and Area properties, Nets, Area and Volume of Solids, Trigonometry, Vectors, Matrix transformations, Lines and Planes in Three Dimensions

**Table 3:** Annotation stages, procedures and deliverables.

Step	Procedure	Deliverable
Calibration step	Annotators review Bloom’s taxonomy, focusing on higher levels, DoK levels 2–4, Hess matrix, and exemplar items	Shared coding understanding
Pair-wise independent annotation	Two annotators classify each item independently	Initial Bloom and DoK labels
Pair comparison	Labels are compared for agreement	Agreement/disagreement record
Consensus	The third expert resolves the disagreement as a tiebreaker	Final consensus label
Reliability quantification	Compute Cohen’s Kappa and Krippendorff’s Alpha	Reliability Values
Documentation	Record final label, annotator IDs, adjudication status	Audit trail

**Table 4:** Description of NLP-derived features.

Feature	Description	Method/Metric
Lexical_Diversity	Type-Token Ratio (TTR):	unique types / total tokens
AVG_Sentence_Length	Mean Words per Sentence:	tokens/sentence count
Readability	Flesch Reading Ease:	[206.835 − 1.015 × (words/sents) − 84.6 × (syllables/words)]
LSA_InterQuestion_Sim	TF-IDF Cosine Similarity	(corpus pairwise): (mean cosine sim of each item vs all others)
LSA_ContextTask_Sim	TF-IDF Cosine Similarity	(item vs centroid): (each item vs corpus means TF-IDF vector)
Clausal Density	Clausal Density Ratio:	subordinate clause markers per sentence + 1)
Symbol-to-Text Ratio (STR)	Non-Alphanumeric Character Ratio:	symbolic characters / total non-whitespace characters)

**Table 5:** Description of the dataset structure.

Variable_Name	Description	Data_Type
Item_ID	Each item's unique identifier	String
Item	Mathematics full-text scenario	Text
Construct	Lower secondary Mathematics four Mathematical Constructs.	Categorical
Bloom_Level	Bloom’s Taxonomy higher-order Cognitive level.	Categorical
DoK_Level	Webb’s Depth of Knowledge higher level.	Categorical
Concepts	Core mathematical concepts assessed in an item.	Text
Lexical_Diversity	Ratio of unique words to the total number in an item.	Float
AVG_Sentence_Length	Average sentence length within the item.	Float
Readability	Flesch Reading Ease score.	Float
LSA_InterQuestion_Sim	Embedding-based similarity score vs other items	Float
LSA_ContextTask_Sim	Embedding-based similarity score between scenario and task.	Float
Clausal Density	Ratio of clauses to sentence	Float
Symbol-to-Text Ratio (STR)	Ration of Math symbols to text	Float

Employing the principles of constructive alignment, each item was linked to the four constructs identified in the table of constructs presented in Table 2. Using an independent paired-review design, 5 expert annotators conducted constructive alignment and mapped annotations onto assessed concepts, employing mathematical knowledge and reasoning in the tasks. One annotator served as a tiebreaker on a rotating basis each day, and disagreements were resolved by consensus and by reference to the table of constructs and curriculum materials. The resulting structure, consisting solely of items, constructs, and assessed concepts, supports granular item analysis and may enable knowledge graph creation, automated item generation, and construct-aware item retrieval.

### Stage 3: cognitive annotation and reliability assessment

Grounded in established taxonomies, stage 3 characterised each item’s cognitive demand. Bloom's taxonomy [9] and Webb’s depth of knowledge (DoK) [8] were used and operationalised using Hess’s Cognitive Rigour Matrix (CRM) [10] via expert protocol-guided annotation. Bloom’s framework classifies types of cognition, while DoK defines the level of complexity of thinking required rather than the assumed type of cognition. Their combination provided a complementary representation of cognitive processes and task depth through CRM, thereby supporting consistent annotation within an established framework [10]. We aimed to complement difficulty ratings with cognitive complexity, treating the two as complementary rather than interchangeable. This is because difficulty may describe how hard an item is, whereas cognitive complexity specifies the type and depth of reasoning it requires of an item taker. Bloom’s Taxonomy levels Analyse, Evaluate, and Create, and Webb’s Depth of Knowledge Skills and Concepts, Strategic Thinking, and Extended Thinking levels 2 to 4, consistent with Hess’s Cognitive Rigour Framework.

The annotation took place using an independent paired-review design, with the last person serving as a tiebreaker in cases of disagreement. Once again, these were not unquestionable authorities and were rotated in each session; disputed cases were reviewed against our explicit annotation protocol. More importantly, both screening and cognitive annotation were treated as analytically distinct phases to reduce confirmation bias. Screening decision history or the modification status for each was not provided to the item annotators responsible for Bloom and DoK mapping. Items were presented in randomised order and coded against the rubric rather than against the dataset's presumed purpose. To minimise cognitive fatigue and maintain annotation consistency over time, annotation sessions were distributed across multiple sittings and periodically recalibrated using benchmark items, with each annotator working on up to 50 items.

Annotators first completed calibration using a shared coding manual and exemplar items as presented in the annotation protocol. The annotation protocol included logical-consistency constraints derived from Hess’s Cognitive Rigour Matrix. For instance, combinations judged theoretically implausible, such as highly Bloom-level classifications paired with the lowest depth-of-knowledge levels, were flagged for secondary review prior to acceptance. Reliability was quantified using Cohen’s Kappa [12] for pairwise annotations and Krippendorff's alpha [13] for overall agreement and annotation consistency. Inter-annotator agreement values demonstrated acceptable to good agreement across the dimensions as presented in Table 6. To minimise human subjectivity and improve consistency, the annotation protocol detailed clear guidelines, decision rules, and examples. All annotation stages, procedures and deliverables are summarised in Table 3.

**Table 6:** Inter-annotator reliability values.

Dimension	Pairing Structure	Statistic Used	Reliability Coefficient	Interpretation
Bloom-Level Mapping	Two independent pairs + Tie-Breaker	Cohen’s Kappa (pairwise averaged)	0.64	Substantial agreement
Bloom-Level Mapping	Full annotation set	Krippendorff’s Alpha	0.7	Acceptable Reliability
DoK-Level Mapping	Two independent pairs + Tie-Breaker	Cohen’s Kappa (pairwise averaged)	0.64	Moderate agreement
DoK-Level Mapping	Full annotation set	Krippendorff’s Alpha	0.66	Acceptable Reliability
Construct Mapping	Two independent pairs + Tie-Breaker	Cohen’s Kappa (pairwise averaged)	0.72	Acceptable Reliability
Construct Mapping	Full annotation set	Krippendorff’s Alpha	0.81	Good Reliability
Concepts Assessed	Two independent pairs + Tie-Breaker	Cohen’s Kappa (pairwise averaged)	0.65	Moderate agreement
Concepts Assessed	Full annotation set	Krippendorff’s Alpha	0.74	Acceptable reliability

### Stage 4: linguistic and semantic feature profiling

Linguistic features of any item also determine the complexity levels of that item. We derived linguistic and semantic features as indicators of cognitive complexity. These features may not be treated as direct measures of cognition; rather, they provide computational descriptors that can be analysed alongside cognitive labels for assessment modelling, difficulty calibration, and educational AI development. The annotation was done via Python-based natural language processing tools and techniques summarised in Table 4. This stage involved text normalisation, tokenisation, segmentation, and parsing of mathematical symbols to enable the processing of mathematical expressions. We then computed various linguistic features, including Lexical Diversity, derived using the Type-Token Ratio [14]; Average Sentence Length; Readability, derived from Flesch Reading Ease Scores [15]; and Clausal Density. Moreover, we employed embedding-based methods [16] to derive semantic analyses, such as Inter-Question Semantic Similarity and Context–Task Semantic Similarity, to analyse structural and semantic connections among items. Fig. 3 shows combined histograms, with Mean, standard deviation and maximum values also presented in Table 7 for the derived features. Finally, we computed the density of mathematical expressions relative to the amount of text per item using the Symbol-to-Text Ratio (STR). Overall, the resulting linguistic profiles presented machine-readable representations of linguistic and semantic structures that can be utilised for cognitive complexity modelling, assessment analytics, and educational AI applications.

**Table 7:** Scales and interpretation of NLP-derived features.

Feature	Description	Unit / Scale	mean	std	min	max	Interpretation
Lexical_Diversity	Vocabulary variation within an item	0–1 index	0.537	0.083	0.277	0.884	Higher values mean good diveristy
AVG_Sentence_Length	Average number of tokens per sentence	tokens	17.276	5.853	6.540	91.500	Higher values indicate longer or complex sentences
Readability	Text difficulty using standardized readability index	score	60.562	14.236	−41.830	93.130	Higher values indicate easier readability
LSA_InterQuestion_Sim	Embedding-based similarity score vs other items	Cosine similarity	0.020	0.007	0.003	0.045	Higher values indicate stronger semantic coherence among items
LSA_ContextTask_Sim	Embedding-based similarity score between scenario and task.	Cosine similarity	0.145	0.048	0.030	0.315	Higher values indicate stronger semantic coherence between scenario and task.
Clausal Density	Ratio of clauses to sentence	Ratio	1.677	0.394	1.000	4.250	Captures mathematical density of item representation
Symbol-to-Text Ratio (STR)	Ratio of math symbols to text	Ratio	0.078	0.042	0.018	0.374	Higher values show high density of mathematical notations.

### Stage 5: dataset structuring and integration

The final stage of the framework was structuring and integration, in which we consolidated all annotations and derived linguistic features into a reusable, machine-readable dataset. Each item was assigned a unique identifier (Item_Id) as a single record with its Construct, Bloom_Level, DoK_Level, Concepts, Lexical_Diversity, AVG_Sentence_Length, Readability, LSA_InterQuestion_Similarity, LSA_ContextTask_Similarity, Clausal Density and Symbol-to-Text Ratio (STR) as core variables, as shown in Table 5. We standardised the variable names for consistency and interoperability.

To ensure that HiCogMath adheres to the Findable, Accessible, Interoperable, and Reusable (FAIR) Principles, a data dictionary describing all variables, annotation procedures, and feature definitions was developed. The final dataset was in Microsoft Excel (.xlsx) format and exported to Comma-Separated Values (.csv) format. We deposited it in the Mendeley Data repository to facilitate accessibility, reproducibility, and reuse in artificial intelligence research for educational assessment.

## Method validation

CoMAD, as a framework, was validated to determine whether it can consistently produce a cognition-aware assessment dataset, like Uganda’s competency-based mathematics HiCogMath dataset. Our validation focused on ascertaining whether the framework produces cognition-aware, curriculum-aligned cognitive annotations, reliable linguistic feature extraction, integrity of dataset structure, and reusability. The significance of the CoMAD methodological contribution lies in its cognitive annotation protocol. We validated the replicability of the operationalisation of Bloom’s revised taxonomy and Webb’s Depth of Knowledge (DoK) via pair-wise independent expert annotation using Hess’s Cognitive Rigour Matrix. Using predefined, calibrated coding guidelines, the inter-annotator agreement values showed that cognitive complexity can be operationalised systematically and transparently, thereby addressing a common limitation of educational AI datasets.

Likewise, linguistic profiling features were validated using automated consistency checks and manual verification of extracted feature values. Average feature values were inspected for computational validity, expected numerical ranges, and consistency with observable characteristics of assessment items. Moreover, the ranges of the derived linguistic features were within acceptable bounds, ensuring the feasibility of subsequent analysis. Moreover, we checked for missing values, field validation and overall schema consistency, verifying whether all variables were populated. Overall, the results of our validation show that the CoMAD framework can be a reproducible, powerful framework for constructing curriculum-aligned, cognition-aware, and computationally reusable assessment datasets, thereby providing a methodological foundation for educational assessment modelling, curriculum-aware knowledge graphs, retrieval-augmented generation systems, automated item generation, and educational large language model research.

## Limitations

This framework is subject to several limitations. In the first place, the framework relied on well-defined curriculum documentation and the availability of subject-matter experts. This means that the availability of standard curriculum specifications and qualified experts to interpret and map assessment constructs, inherent concepts, and cognitive frameworks determines the framework's success. Additional criteria and procedures may be needed in educational settings where there are few experts, and curriculum specifications are not well defined. Moreover, while the sourcing method ensures cultural and contextual alignment and construct validity, it limits the generalizability of the dataset's use cases across other STEM domains, educational systems, curricula, or linguistic contexts, given its focus on mathematics alone. Additionally, the dataset exclusively focused on the creation of higher-order thinking items. Therefore, this does not represent the full Bloom and DoK spectrums of cognitive complexity required for mathematics assessment. Investigations requiring all levels may require new datasets. And if applications are required for use cases that emphasise factual recall, informal learning tasks, or competency structures that are not readily represented by integrated assessment constructs, then the mapping and annotation criteria must be modified.

Instead of empirically calibrated item difficulty, the cognitive labels in CoMAD reflect expert-judged cognitive demand informed by established cognitive taxonomies. Cognitive complexity and difficulty are two different but related concepts. When learner response data, pass rates, or item response theory parameters become available, future validation should connect them with CoMAD labels. Because the dataset was specifically linked with the Ugandan curriculum, the annotation procedure depended on seasoned maths teachers from Uganda. However, systemic bias may be introduced by localised educational interpretations. External reviewers from different educational systems should be included in future validation efforts, and classifications should be benchmarked against global evaluation frameworks such as Trends in International Mathematics and Science Study (TIMSS) and Programme for International Student Assessment (PISA).

Taxonomy-based annotations provide unique structured proxies but merely infer expected cognitive reasoning rather than psychometric learner cognition. Moreover, the annotation depends on expert judgement and inter-annotator agreement. While these methods are reliable, annotators may need to adapt or be trained to handle unfamiliar subject matter or may struggle with complex workloads as dataset sizes increase. Additionally, the items were primarily textual, eliminating the visual content that is paramount for interpreting mathematical problem contexts. Thus, a change in modality may affect certain procedural components in the framework. Despite the use of multiple expert annotators and the quantification of reliability, this method is insufficient to eliminate residual subjectivity. Finally, while the methodology is designed to be transferable to other educational contexts or subject domains, adapting it to new subjects and contexts may require developing unique annotation guidelines, feature extraction procedures, and validation criteria. An educational context, assessment standards and procedures, and subject characteristics may be determining factors for the needed adaptation.

## CRediT author statement

**Tibakanya Joseph:** Conceptualisation, Methodology design, Data collection and curation, Writing, Original draft preparation, and Writing- Reviewing. **Joyce Nakatumba-Nabende**: Supervision, Conceptualisation, Methodology design, and Writing- Reviewing. **Agaba Joab Ezra:** Methodology design, Writing, and Reviewing. **Ampeire Henry Kariisa:** Methodology design, Writing, and Reviewing.

## Ethics statements

All content in this methodology, including the HiCogMath dataset, is sourced exclusively from publicly available materials. As such, no personally identifiable details are included, and no sensitive learner data is collected during the process. Additionally, the dataset creation process does not involve any human subjects; it is derived from existing or formulated artefacts used in formal assessment environments.

Expert annotators involved in the curation process did so through voluntary engagement. No personal or sensitive details were collected from them or their learners. The annotation process focused solely on them, judging the assessed concepts, item complexity, and construct classification.

Core emphasis was placed on academic integrity and on responsible, robust methodologies for dataset creation and curation to ensure ethical reuse. Responsible data governance, including fairness in representation, transparency in processing, and respect for intellectual property. The methodology and dataset are strictly intended for research involving computational analysis, cognition modelling via machine learning, automated item generation, and LLM alignment. Thus, no attempt has been made to link the methods and dataset content to individual educators, schools, or learners, thereby ensuring anonymity and protecting privacy.

## Declaration of AI-Assisted technologies in the writing process

During manuscript preparation, an artificial intelligence tool, specifically Grammarly, was used exclusively for language editing. After using these tools, the author reviewed and edited the content as needed and takes full responsibility for the publication's content.

## Declaration of competing interest

The authors declare that they have no known competing financial interests or personal relationships that could have appeared to influence the work reported in this paper.

## Data Availability

HiCogMath, a dataset produced durong the method validation has been published and the link is provided in the manuscript.
